# The Inverse Spacer—A Novel, Safe, and Cost-Effective Approach in Routine Procedures for Revision Knee Arthroplasty

**DOI:** 10.3390/jcm10050971

**Published:** 2021-03-02

**Authors:** Kristoff Hammerich, Jens Pollack, Alexander F. Hasse, André El Saman, René Huber, Markus Rupp, Volker Alt, Raimund W. Kinne, Joerg Mika

**Affiliations:** 1 Department of Orthopaedic Surgery, Eichsfeld Klinikum gGmbH, Academic Teaching Hospital of the University of Goettingen, 37308 Heilbad Heiligenstadt, Germany; k.hammerich@gmx.net; 2 Department of Orthopaedic Surgery, SRH Wald-Klinikum Gera, Academic Teaching Hospital of the University of Jena, 07548 Gera, Germany; jens.pollack@srh.de; 3 Department of Internal Medicine 1, Section of Hematology/Oncology/Rheumatology, University Hospital Homburg, 66421 Homburg, Germany; alexanderhasse@me.com; 4 Department of Trauma, Hand and Reconstructive Surgery, University Hospital Frankfurt, 60590 Frankfurt am Main, Germany; andre.elsaman@kgu.de; 5 Experimental Rheumatology Unit, Department of Orthopedics, Jena University Hospital, Waldkliniken Eisenberg GmbH, 07607 Eisenberg, Germany; huber.rene@mh-hannover.de (R.H.); raimund.w.kinne@med.uni-jena.de (R.W.K.); 6 Institute of Clinical Chemistry, Hannover Medical School, 30625 Hannover, Germany; 7 Department of Trauma Surgery, University Hospital Regensburg, 93053 Regensburg, Germany; markus.rupp@ukr.de (M.R.); office.uch@ukr.de (V.A.)

**Keywords:** inverse spacer, intraoperatively molded, cost-effective, (sub-) luxation, dislocation, revision knee arthroplasty

## Abstract

Background: A major disadvantage of current spacers for two-stage revision total knee arthroplasty (R-TKA) is the risk of (sub-) luxation during mobilization in the prosthesis-free interval, limiting their clinical success with detrimental consequences for the patient. The present study introduces a novel inverse spacer, which prevents major complications, such as spacer (sub-) luxations and/or fractures of spacer or bone. Methods: The hand-made inverse spacer consisted of convex tibial and concave femoral components of polymethylmethacrylate bone cement and was intra-operatively molded under maximum longitudinal tension in 5° flexion and 5° valgus position. Both components were equipped with a stem for rotational stability. This spacer was implanted during an R-TKA in 110 knees with diagnosed or suspected periprosthetic infection. Postoperative therapy included a straight leg brace and physiotherapist-guided, crutch-supported mobilization with full sole contact. X-rays were taken before and after prosthesis removal and re-implantation. Results: None of the patients experienced (sub-) luxations/fractures of the spacer, periprosthetic fractures, or soft tissue compromise requiring reoperation. All patients were successfully re-implanted after a prosthesis-free interval of 8 weeks, except for three patients requiring an early exchange of the spacer due to persisting infection. In these cases, the prosthetic-free interval was prolonged for one week. Conclusion: The inverse spacer in conjunction with our routine procedure is a safe and cost-effective alternative to other articulating or static spacers, and allows crutch-supported sole contact mobilization without major post-operative complications. Maximum longitudinal intra-operative tension in 5° flexion and 5° valgus position appears crucial for the success of surgery.

## 1. Introduction

Predominant causes for revision total knee arthroplasty (R-TKA) are periprosthetic joint infections (PJI; 22.6%), followed by instability (20.0%), aseptic loosening (14.9%), and retropatellar arthrosis (14.2%; [1]). Prevalence and resistance pattern of the micro-organisms involved in PJI, as well as the empirical antibiotic approach differ among countries and hospitals [2,3]. While acute infection (<4 weeks) can usually be treated by debridement, antibiotic therapy, and implant retention (DAIR), gold standard therapy of chronic infection regularly requires two-stage R-TKA [4,5].

The first stage of R-TKA consists of removal of the old prosthesis, aggressive tissue debridement, and insertion of antibiotic-loaded static or articulating spacers [6,7]. The main functions of these spacers are: (1) placeholder action to avoid shortening of muscles and connective tissue by maintaining tissue pre-tension; (2) joint stabilization to avoid luxation; (3) reduction of dead space to limit hematoma formation and/or proliferation of connective and fat tissue; (4) local delivery of antibiotics; and, in the case of articulating spacers, (5) preservation of joint mobility to avoid stiffness and support a better postoperative range of motion (ROM) and functional outcome ([8] and references therein). The second stage is the implantation of a new prosthesis in the absence of hematological, joint aspirate, or clinical signs of infection [7].

Static spacers, on one hand, are inexpensive and manually molded from polymethylmethacrylate (PMMA) bone cement during R-TKA. They aim at achieving optimal adaptation to existing bone surface and joint space in patients with ligamentous instability and large defects after explantation. Due to their design, static spacers resemble an arthrodesis with limited mobility of the knee joint and the potential for increased bone loss. This may either lead to joint stiffness and subsequent problems upon re-implantation [9,10,11] or fractures of spacers or bone and subsequent spacer dislocations upon excessive stress in the prosthesis-free interval [11].

Articulating spacers, on the other hand, were primarily developed to improve the mobility in the prosthesis-free interval [12,13,14] and therefore to prevent joint stiffness and related complications [15,16]. There are numerous designs of different articulating and non-articulating spacers, including completely hand-made, mold-supported, commercially preformed/customized shapes or even surgical approaches based on removed prostheses following sterilization [16,17,18], and various combinations of the above. Contrary to initial expectations based on improved knee motion after re-implantation, static and dynamic spacers did not significantly differ in pain, functional scores, bone loss, or re-infection rates, leading to the suggestion to “consider both static and articulating spacers in the treatment of infection following total knee arthroplasty and to tailor treatment on the basis of patient-related factors” [7,13,19,20,21]. The clinical application of such articulating spacers is however limited by the size of the defect, for example in tumor surgery. The high cost of the different spacers depends on material cost, personnel input, and time in the operating room and represents a strong incentive to develop cost-effective, but safe new spacer designs [16].

Therefore, the aim of this study was to develop and evaluate the results of such a novel spacer without the risk of (sub-) luxation, paying particular attention to: (1) a fine-tuned shape of the spacer (for the first time inverted with a convex tibial and a concave femoral component); (2) joint positioning during the final molding and hardening of the spacer (maximum longitudinal tension in 5° flexion and 5° valgus position); (3) defined mechanical conditions during the post-operative mobilization of the patient (straight leg brace; sole contact mobilization with crutches and limited ROM).

## 2. Materials and Methods

### 2.1. Patient Cohort

The Caucasian patient cohort in this retrospective study (for Institutional Review Board Statement see below) consisted of all 101 chronic PJI patients admitted to the Department of Orthopedics, Jena University Hospital, Waldkliniken Eisenberg GmbH during a period of 6 consecutive years (2008–2013; 46 males, 66.4 ± 1.4 years (mean ± SEM), range 43 to 82 years; 55 females, 67.9 ± 1.2 years, range 52 to 89 years) without any exclusion criteria. The diagnosis of the PJI was based on internationally established procedures including a combination of laboratory, histopathology, microbiology, and imaging studies ([5] and references therein). In these patients, a total of 110 R-TKA were performed, in 2 cases bilateral and in 7 cases repeated R-TKAs of the same joint. The mean service life of the most recently implanted prostheses was 41.0 ± 3.4 months (min. 1 month, max. 240 months). The clinical diagnosis of all patients before the original TKA was either primary or secondary osteoarthritis (in the latter case without any details on the underlying disorder). The patients were coded with four different International Classification of Procedures in Medicine (ICPM) keys concerning the removal of a bicondylar surface replacement prosthesis (ICPM 5-823.7, 99 cases), removal of a hinge prosthesis (ICPM 5-823.8, 8 cases), removal of a special prosthesis (ICPM 5-823.a, two cases), or removal of a prosthesis without specification (ICPM 5-823.y, 1 case).

### 2.2. Spacer Design

**Figure 1:**
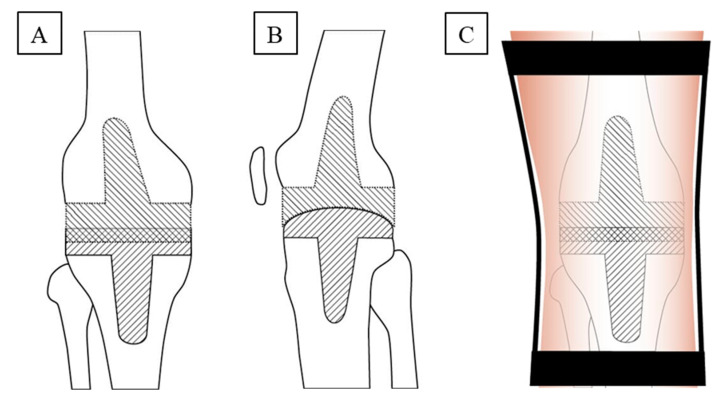
Spacer design and mechanical conditions during post-operative mobilization of the patient. (**A**) Anterior to posterior and (**B**) lateral, schematic design of the inverse spacer, consisting of convex tibial and concave femoral components, each equipped with a stem component to ensure optimal anchoring in the medullary canal for rotational stability; (**C**) application of a straight leg brace to prevent medio-lateral knee movement and strongly limit knee flexion. The spacer design combines the following features: (i) tight embracing of the convex tibial part by the concave femoral part in a hinge-like joint sufficiently prevents anterior to posterior spacer translation; (ii) smaller bone defect, anatomic shape, and a levelled, broad weight-bearing surface suggested a convex tibia component, a larger, anatomically concave femur defect with a central notch suggested a concave femoral component; (iii) successive molding/polymerization of the tibial and femoral components under maximal tension allowed adaptation of the two components with minimized play.

For the first time, the present spacer was designed as a stem-reinforced, inverse spacer with a convex tibial and a concave femoral component (Figure 1A,B).

The advantages of this design are the following: (i) tight embracing of the convex tibial part by the concave femoral part recalls pure hinge joints such as the elbow joint, in which approximately 50% of the convex trochlea is covered by the concave ulna. This shape sufficiently prevents anterior to posterior (ap) translation of the spacer; (ii) the usually smaller bone defect on the tibia, in conjunction with its anatomic shape and its levelled, broad weight-bearing surface, suggested a convex shape on the tibia. In contrast, the larger, anatomically concave femur defect with the central notch rather suggested a concave shape of the femoral component; (iii) after initial molding and polymerization of the tibial component, molding of the concave femoral component under maximal tension turned out straightforward and efficient, allowing to adapt the two spacer components with minimized play until the last minute of the polymerization. In addition, both parts of the spacer were designed with a stem component to ensure optimal anchoring in the medullary canal for rotational stability [11,14].

The novel inverse spacer was intraoperatively molded with PMMA bone cement containing antibiotics adapted to the antibiogram of the respective microorganism according to the supplier’s instructions (Heraeus Medical, Wehrheim, Germany; mostly pre-fabricated gentamicin and/or clindamycin palacos—both 1 g/40 g of palacos—or, alternatively, self-prepared vancomycin palacos—≤4 g/40 g palacos—). For this purpose, first the tibial spacer component was pre-formed in the surgeon’s hand, implanted, and formed in a convex manner with a spatulum. Thereafter, the femoral spacer component was pre-formed, implanted, and intra-articularly molded with a spatulum to adapt its concavity as closely and congruently as possible to the convex tibial component without specific adaptation to the retropatellar surface (for details of the joint position see below). Comparable amounts of PMMA bone cement were utilized for the two different spacer components.

The cement was molded during the bonding phase to avoid complete bony integration, but reach sufficient bony anchorage. This allowed a quicker removal without causing more defects before the re-implantation. To maximize the surface for the release of antibiotics, the cement was mixed without vacuum.

### 2.3. Surgery

After removal of the infected prosthesis and tissue sampling, a wide surgical debridement and complete synovectomy was performed. This was followed by irrigation of the joint with sterile, iso-osmotic LAVANID^®^ ½ polyhexanide wound solutions for 15 min (SERAG-WIESSNER GmbH & Co. KG, Naila, Germany), rinsing with 0.9% saline solution (B. Braun Melsungen AG, Melsungen, Germany), and implantation of the inverse spacer as described above. During implantation of the femoral component, the leg was stringently held by an assistant in 5° flexion and 5° valgus position under maximum longitudinal tension, a step considered crucial for successful spacer molding and implantation. After spacer implantation and full polymerization, an intra-articular drain was inserted, followed by conventional closure of the situs and sterile wound dressing. All patients received intravenous antibiotic therapy according to the concurrent international guidelines for 10 days with subsequent oral antibiotic coverage for a total of four weeks, followed by an antibiotic holiday until re-implantation.

Anterior to posterior and lateral X-rays of the knee were taken before and after prosthesis removal and re-implantation.

### 2.4. Postoperative Mobilization

Postoperative crutch- or walker-assisted mobilization with obligatory application of a straight 0° leg brace (Figure 1C) was limited to sole contact and took place under the guidance of a physiotherapist. Static muscle and tension training was performed, including the adjacent joints on the operated side and the healthy contralateral joints. If the aspirate from the knee puncture 6 weeks after explantation showed a negative microbiological result, re-implantation was performed eight weeks after prosthesis removal. The patients were then discharged if: (i) they were comfortably walking with assistive devices; and (ii) the ROM of the operated knee reached 0°–0°–90° (extension/flexion).

The Kaplan–Maier failure rate of the spacer was calculated as previously published [22].

## 3. Results

All patients with a diagnosed or suspected infection after TKA during a period of six consecutive years (*n* = 101; total of 110 R-TKAs) were treated with the inverse antibiotic- loaded spacer. In 3 patients, an early spacer exchange was necessary within the first week after the primary spacer implantation because of persistent or increased infection parameters and continuing wound secretion. The X-ray after spacer implantation indicated a correct positioning of the spacer without fractures of spacer or bone.

**Figure 2:**
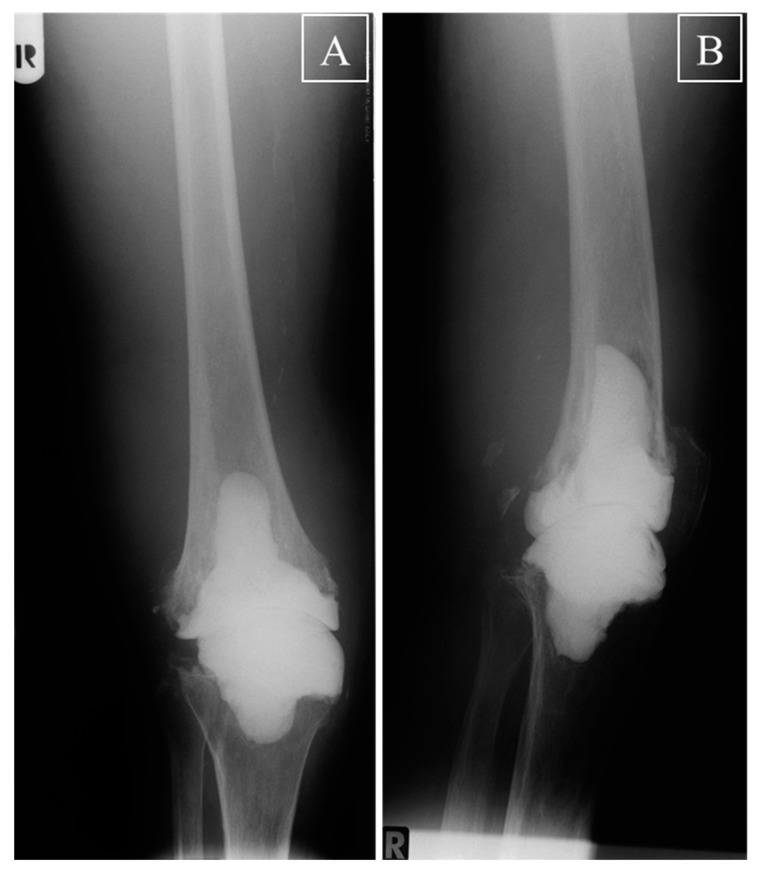
Knee X-ray before re-implantation. (**A**) Anterior to posterior and (**B**) lateral X-ray of a right knee with the inverse spacer of one representative patient before re-implantation TKA. A perfect fit of the two different spacer components into the tibial and femoral medulla was observed, without any signs of dislocation, subluxation, fractures of spacer or bone, or increased gross abrasion of bone in the joint area; R = right.

Early mobilization under sole contact with crutches or other devices was excellently tolerated by all patients without clinical signs of dislocations, subluxations, fractures of spacer or bone, or soft tissue compromise requiring reoperation. X-rays taken before re-implantation also did not show any indications for dislocation, subluxation, fractures of spacer or bone, or increased gross abrasion of bone in the joint area in any patient (Figure 2A,B), resulting in a calculated Kaplan–Maier failure rate of zero for the spacers.

After a prosthesis-free interval of 8 weeks or, in the case of early spacer exchange, nine weeks, all patients were successfully re-implantated without any complications.

## 4. Discussion

The present study reports on a novel, hand-made, stem-reinforced inverse spacer, consisting of a convex tibial and a concave femoral component of PMMA bone cement, which was intra-operatively molded under maximum longitudinal tension in 5° flexion and 5° valgus position. R-TKA with this spacer in 110 knees with diagnosed or suspected PJI, with subsequent application of a straight leg brace and crutch-supported full sole contact, resulted in early mobilization of the patients without any clinical or X-ray signs of dislocations, subluxations, fractures of spacer or bone, or soft tissue compromise requiring re-operation. This novel approach resulted in a Kaplan–Maier failure rate of zero for the spacers and may thus represent an attractive, safe, and cost-effective alternative to other spacers presently used for R-TKA. Detailed characterization of the biomechanical properties of the novel inverse spacers remains the objective of further studies [18].

The present study for the first time describes a novel spacer design with a convex tibial and a concave femoral component. On one hand, the combination of the present articulating spacer and the straight leg brace rather makes it functionally act like a static spacer, although it was in principle designed as an articulating spacer with independent tibial and femoral components. One reason is the restriction of the ROM of the resulting artificial “joint” due to the resemblance with a pure hinge joint, basically eliminating anterior to posterior translation of the spacer. In addition, flexion and/or lateral movement of the knee was largely restricted by the straight leg brace. For this reason, the success rate of the present spacer may be best comparable to the respective rates of other static spacers. Indeed, Voleti et al. reported a very low complication rate of 1% for static spacers ([7]; and references therein) and Johnson et al. even described 0% complications [11], in good agreement with the results of the current study. However, there are also reports showing a rate of up to 15% mechanical complications after the use of static spacers for R-TKA [23].

On the other hand, the brace does not completely eliminate movement within the spacer, allowing a limited degree of flexion and lateral movement. This minimizes the risks of spacer or bone fractures usually associated with the use of purely static spacers. It also addresses and supports the delicate balance between complete immobilization to favor eradication of the infection and sufficient residual movement to partially reduce stiffening of the knee joint during the prosthesis-free interval. The present results can thus also be compared with previous reports on the use of articulating spacers for R-TKA. In this aspect, the current spacer shows a very favorable, low complication rate of 0%, whereas previous reports demonstrate an increased complication rate between 3% and 57% for articulating spacers [7,11,24]. In the latter publication, these rates included numerous mechanical complications, i.e., 24% component tilting, 21% medio-lateral translations, 3% dislocations of tibial or femoral components, 5% fractures of the femoral spacer component, and 4% of manifest anteroposterior joint subluxations [24]. Thus, the low complication rates of the present spacer appear very encouraging, possibly due to its final in situ performance as a static spacer during two-stage R-TKA.

In the present study, the femoral spacer component was not specifically adapted to the retropatellar surface. The authors would like to point out, however, that a physiological function of the patella may not have been necessary due to the restricted movement of the knee during the prosthesis-free interval, and that a satisfactory ROM of the operated knee of 0°–0°–90° (extension/flexion) was still reached in all patients before re-implantation.

The advantages of the current two-component spacer with restricted post-operative ROM in comparison with a conventional static spacer can thus be summarized as follows: (i) by reducing the post-operative forces/friction occurring at the cement-spacer interface, the current spacer design may help to avoid gross bone loss and bone fractures, as exemplified in representative Figure 2; (ii) in comparison to a large, more bulky one-component static spacer, the two segments of the current spacer appear to simplify and optimize the preparation and implantation of the spacer, including a more convenient sequential pre-forming, insertion, molding, and hardening of the separate components under conservation of the appropriate joint length and tension; (iii) the current spacer may allow an easier and less time-consuming removal of the spacer during re-implantation TKA. A spacer design with a convex femoral component and a concave tibial component with a higher anterior and posterior lip may also be suitable to avoid anterior or posterior luxation, but may be more susceptible to damage by high axial forces acting on the spacer.

Of note, Lanting et al. pointed out that sagittal (anterior-posterior), but not coronal (medio-lateral) subluxation, of the knee had a significant influence on early to mid-term functional knee scores following a two-stage R-TKA for infection [8]. This is in clear agreement with the notion that the current spacer, due to its hinge-like design, strongly limits anterior-posterior movement and provides yet another argument in favor of the present spacer design.

Standardized fabrication of spacers for R-TKA may be of advantage to favor reproducible spacer shapes and features and in order to save operating time and personnel effort. Indeed, there are several studies reporting on pre-fabricated spacer components or molds for standardized production of spacer parts with PMMA bone cement [11,17,18]. However, the current, purely hand-made spacer allows both straightforward and efficient molding and optimal adaptation of each spacer component to the existing bone defect until the last minute of polymerization. This may possibly provide optimized conditions for a successful prosthesis-free interval without major complications [7,11,24].

In the present study, maximum longitudinal intra-operative tension along the physiological axis in 5° flexion and 5° valgus position proved crucial for the success of surgery. This was necessary to position the spacer with minimized play in the artificial “joint” and thus to avoid initial wobbling possibly leading to loosening of the spacer. This concept is in good agreement with the statements of other authors, who suggested to achieve the correct, quasi-physiological tension in the knee joint by using appropriately sized components and/or cement mantle thicknesses, thus ensuring that the implant is neither fitted too loosely nor too tightly [9,11,15,25]. If such optimal fitting was not possible, the authors suggested the application of a static spacer [11]. However, the joint tension generated in the present R-TKA procedure clearly exceeds the quasi-physiological tension suggested by others. This represented an attempt to stabilize the joint by allowing optimized adaption of the two spacer components with minimized play in the intra-spacer joint. The authors strongly believe that this detail may be pivotal for the success of the present procedure.

The application of a straight leg brace was also considered crucial for the clinical success, since it prevented medio-lateral knee movement and strongly limited knee flexion. In the previous literature numerous different possibilities for the regulation of joint ROM were applied, ranging from post-operative mobilization without any brace for dynamic spacers [11] to complete immobilization of the knee with a temporary cast for a few days [15,16], a mobile brace [20], or a straight leg brace for at least three to four weeks [26]. On the basis of the critical situation for the final clinical success of R-TKA, the authors would presently recommend to stay with the application of the straight leg brace and to rather consider slight modifications of the joint loading under the protection of the leg brace to foster physiological movement and thrombosis protection.

In line with these considerations, crutch-supported sole contact mobilization was considered appropriate in the present clinical design of the R-TKA. As in the case of the brace application, several different approaches for weight bearing have been reported previously, ranging from sole contact (present study), to partial or “gentle” weight bearing [11,15,20], and full weight bearing [16]. As mentioned above, slight modifications of the joint loading under the protection of the leg brace may be considered, however, without increasing the loading to a degree augmenting bone loss by spacer-bone friction. Of note, it has to be considered that the patient compliance to restrict the weight bearing to sole contact or the respective loading modification may be limited.

## 5. Limitations of the Study

The present study has the following limitations: (i) it contains a limited number of R-TKAs (total of 110), however, it is one of the few studies with a case number clearly exceeding 100 (e.g., [4,7,8,9,24]); (ii) the effective bone loss at the end of the prosthesis-free interval or the long-term clinical outcome after re-operation remains to be determined; (iii) a control group with (an)other spacer(s) is not included; (iv) data cannot be provided on the biomechanical characterization of the spacer [18] or gait analysis in the prosthesis-free interval, potentially including movement sensors for the spacer.

## 6. Conclusions

The study confirms our main hypothesis that the novel, correctly implanted inverse spacer, in conjunction with our routine clinical procedure, seems to be a safe and cost-effective alternative to other articulating or static spacers for the therapy of PJI after primary TKA, allowing crutch-supported sole contact mobilization without major post-operative complications. Whether the spacer is also suitable for partial or full weight bearing, as possibly and unintentionally performed in the present study, will have to be analyzed in future studies.

## Data Availability

The data presented in this study are available on request from the corresponding author.
